# Generalized Reversible Data Hiding with Content-Adaptive Operation and Fast Histogram Shifting Optimization

**DOI:** 10.3390/e23070917

**Published:** 2021-07-19

**Authors:** Limengnan Zhou, Hongyu Han, Hanzhou Wu

**Affiliations:** 1 School of Electronic and Information Engineering, Zhongshan Institute, University of Electronic Science and Technology of China, Zhongshan 528402, China; dreamzlmn@gmail.com; 2 School of Computer Science, Sichuan Normal University, Chengdu 610000, China; hongyuhanswjtu@163.com; 3 School of Communication and Information Engineering, Shanghai University, Shanghai 200444, China

**Keywords:** reversible data hiding, watermarking, dynamic prediction, histogram shifting, optimization

## Abstract

Reversible data hiding (RDH) has become a hot spot in recent years as it allows both the secret data and the raw host to be perfectly reconstructed, which is quite desirable in sensitive applications requiring no degradation of the host. A lot of RDH algorithms have been designed by a sophisticated empirical way. It is not easy to extend them to a general case, which, to a certain extent, may have limited their wide-range applicability. Therefore, it motivates us to revisit the conventional RDH algorithms and present a general framework of RDH in this paper. The proposed framework divides the system design of RDH at the data hider side into four important parts, i.e., binary-map generation, content prediction, content selection, and data embedding, so that the data hider can easily design and implement, as well as improve, an RDH system. For each part, we introduce content-adaptive techniques that can benefit the subsequent data-embedding procedure. We also analyze the relationships between these four parts and present different perspectives. In addition, we introduce a fast histogram shifting optimization (FastHiSO) algorithm for data embedding to keep the payload-distortion performance sufficient while reducing the computational complexity. Two RDH algorithms are presented to show the efficiency and applicability of the proposed framework. It is expected that the proposed framework can benefit the design of an RDH system, and the introduced techniques can be incorporated into the design of advanced RDH algorithms.

## 1. Introduction

Reversible data hiding (RDH) [1,2], also called reversible watermarking (RW), is referred to as the art of embedding extra data, such as source information and authentication data, into a host signal (also called cover) by slightly modifying the host signal. The embedded information and the original host signal can be fully reconstructed from the marked content by a legal receiver [3,4]. As RDH enables us to perfectly recover the original host content, it is quite desirable and helpful in some sensitive scenarios, such as medical image processing, remote sensing, and military communication.

Up to now, a number of RDH techniques have been reported in the literature. Early methods [5,6] mainly use lossless compression (LC) techniques to substitute a part of the host with the compressed code of the substituted part and the secret message. Since the LC procedure is often applied to the noise-like component of the host, the introduced distortion due to data embedding can be kept low. However, as the entropy of the noise-like component of the host is very high, the compression rate will be very low, which indicates that the (pure) embedding capacity of LC-based RDH is low. More efficient RDH methods are thereafter designed to increase the embedding capacity, e.g., difference expansion (DE) [1,7] and histogram shifting (HS) [2]. Since better payload-distortion performance can be achieved by exploiting the prediction-errors (or differences) of cover elements, various RDH techniques [8,9,10,11,12] have been developed along this line. It can be said that, today, most RDH methods use prediction-errors (PEs) of cover elements to hide secret data since data embedding in PEs can always provide superior payload-distortion performance. Though these algorithms differ from each other in terms of the working mechanism, they are essentially finding the most compressible component of the host (i.e., the noise-like component with the minimum entropy) so that high capacity or low distortion can be achieved.

In PE-based RDH methods, there are two important steps: content prediction and data embedding. The former can be separated into two stages. First, some cover elements are selected out in advance. Then, the others are orderly predicted to generate a PE histogram (PEH). In the first stage, the pre-selected cover elements are usually unchanged throughout the content prediction, ensuring that both the data hider and the data receiver can find the identical prediction values. In the second stage, a suitable predictor is required to obtain accurate estimation of the cover elements to be embedded. The existing works [10,12] often use fixed content pre-selection rule and predictor, which, actually, is not desirable in applications according to the Kerckhoffs’s principle since one may successfully reconstruct the marked PEH.

For data embedding, HS [2] is still the most common operation in today’s RDH techniques. Variants, such as prediction-error expansion (PEE) [13], have also been developed. In HS, one hopes to select such PEH bins that the required payload can be carried by shifting the PEH bins while the distortion is as low as possible. The traditional methods often empirically tune the shifting parameters, which is not applicable in practice. And, as a host signal can be utilized to embed the secret data several times, it is actually time consuming for searching parameters and makes a it hard for a reader to reproduce the simulation results due to this non-deterministic empirical operation.

Therefore, on the one hand, it is quite desirable to design a general framework for RDH so that many existing RDH works can be generalized and more advanced RDH schemes (in terms of the payload-distortion performance and the security) can be developed based on the designed framework. On the other hand, by optimizing the data embedding parameters in a deterministic and fast manner, the RDH systems will be more applicable to practice. In order to fill this research gap, in this paper, we revisit the conventional RDH algorithms and propose a general framework of RDH. Meanwhile, we propose a fast and efficient parameter optimization algorithm for HS-based embedding. Two RDH methods based on the proposed framework are further introduced to demonstrate the superiority and applicability of the proposed work. In summary, the main contributions of this paper can be described as follows:

- We propose a general framework dividing the PE-based RDH design into four parts so that we can easily design or improve an RDH system. The four parts are named as binary-map generation, content prediction, content selection, and data embedding. To ensure the security, we use a secret key to control the binary-map generation, and a dynamic predictor for content prediction. For content selection, we use a local-complexity evaluation function to preferentially use smooth elements.
- We propose a fast histogram shifting optimization algorithm to determine the near-optimal embedding parameters for HS-based RDH. A significant advantage is that the embedding performance can be kept sufficient, while the computational cost is low.
- We present two detailed RDH methods to demonstrate the generalization ability of the proposed framework. Extensive experiments are also conducted to verify the superiority and applicability of the proposed work.

The rest of this paper is organized as follows. The proposed framework is first introduced in Section 2. Then, we study each part of the introduced framework in Section 3. Thereafter, in Section 4, two RDH algorithms and experimental results are presented to demonstrate the efficiency and applicability of the proposed work. Finally, we conclude this paper in Section 5. This work extends [14,15] to a general case.

## 2. Sketch of Proposed Framework

**Figure 1:**
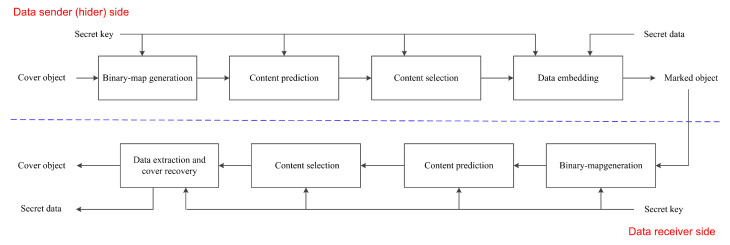
Sketch for the proposed framework. For the data hider, there are four key steps, i.e., binary-map generation, content prediction, content selection, and data embedding. For the data receiver, the key steps include binary-map generation, content prediction, content selection, data extraction, and cover recovery.

As shown in Figure 1, the proposed framework (at the data hider sider) has four parts: binary-map generation, content prediction, content selection, and data embedding. The binary-map generation produces a binary matrix with the same size of the cover, in which “0”s are elements used for content prediction or storing parameters, and “1”s are elements to be probably embedded. The proposed framework does not specify the type of the cover. It indicates that both the binary matrix and the cover elements could be arbitrary dimensional. Unless mentioned, we use a grayscale image as the cover, i.e., one may imagine that the binary matrix is 2D, and the elements are corresponding to image pixels.

The content prediction enables us to use elements marked as “0” to predict those marked as “1”. In this way, the PEs of elements marked as “1” can be obtained. The PEs will be used to carry a payload. The PEs are noise-like, which would not introduce obvious artifacts. After content prediction, one may use a content selection method to preferentially use the cover elements that can benefit the payload-distortion performance. For example, smooth pixels in an image will be preferred for RDH since their PEs are often smaller than the PEs of complex pixels. Finally, HS or its variants can be used for data embedding. Though some methods may not use HS directly, they can also be generalized by this framework. In the following, we revisit typical RDH algorithms and point out that they can be generalized by this framework.

### 2.1. LC-Based RDH

We analyze the simplest case that uses lossless compression. The pixels in an image are divided into two sets $S_{0}$ and $S_{1}$, where $|S_{0}\left|<<\right|S_{1}|$. The LSBs of pixels in $S_{0}$ will carry the system parameters, such as the key. The LSBs of pixels in $S_{1}$ are losslessly compressed. The compressed code, original LSBs of $S_{0}$, and secret data are embedded into $S_{1}$ by LSB substitution. $S_{0}$ can be marked as “0” and $S_{1}$ for “1”. Though there is no intuitive content prediction process, all prediction values can be treated as zero. The data hider can use a key to control the embedding order, which is equivalent to content selection. The LSB substitution is a special case of HS. Clearly, we first empty the LSBs of $S_{1}$. The prediction values of $S_{1}$ are all zero. Thus, if we only use the LSBs of $S_{1}$, the corresponding PEH has only occurrence at zero bin. By shifting “0” to “0/1”, the secret bits can be embedded.

### 2.2. Ni et al’s Method

Ni et al. [2] use the bin-pairs of the histogram directly determined from an image. Though there are not intuitive binary-map generation, content prediction, and content selection, one can mark a part of pixels as “1” and consider their prediction values as zero. To hide secret data, we self-embed some parameters into specific pixels. The LSBs of these pixels are replaced by the parameters, and the original LSBs are recorded as a part of the secret data. These pixels are unchanged in subsequent procedure. They could be marked as “0” in the binary-map. Thus, Ni et al.’s method can be generalized by the proposed framework.

### 2.3. Tsai et al.’s Method

Tsai et al. [8] divide an image into disjoint blocks. The central pixel for each block serves as the prediction of adjacent pixels within the block. The central positions of all blocks are marked as “0”, and the others are “1”. Though there has no intuitive content selection process, one can randomly generate a value for each pixel so that the pixels can be orderly embedded with the HS operation.

### 2.4. Sachnev et al.’s Method

In Reference [10], the authors divide the pixels into two sets named as dot set and cross set. The pixels in the dot set are used to predict that in the cross set, which are thereafter used for data embedding. In this case, one can set the dot-pixel-positions as “0” and the others as “1”. Then, with their introduced local-complexity function, the data hider can select relatively smooth pixels for RDH using the HS. When the dot pixels are used for data embedding, the process is similar. Sachnev et al.’s work exactly matches our framework.

### 2.5. Transformed Domain-Based RDH

With an image, we may not embed data in the spatial domain, but embed data in the transformed domain. In this case, we may determine the binary-map in the transformed domain. We can adjust the boundary pixels into the reliable range in the spatial domain in advance so as to avoid the underflow/overflow of pixels. Thereafter, the content prediction, content selection, and data embedding are applied to the transformed domain.

### 2.6. Expansion-Based RDH

With a prediction-error or difference *d*, we may not shift *d* to d+1 or d−1 to carry a message bit, but expand *d* to 2d and replace the LSB with the secret bit. This operation (called expansion) can be treated as a variant of HS, in that we are actually shifting *d* to 2d or 2d+1, which looks like a “jump”. Therefore, some expansion-based RDH algorithms can be described by the proposed framework, as well.

It can be inferred that our framework can generalize many RDH systems. It is quite helpful in practice since one can easily design, implement, and improve an RDH system. We will give detailed descriptions and analysis in the subsequent sections.

## 3. Details of Proposed Framework

In this section, we analyze each part of proposed framework in detail, and reliable techniques are introduced to achieve better performance. Furthermore, relationships between different parts and other perspectives are provided for better generalization.

### 3.1. Binary-Map Generation

**Figure 2:**
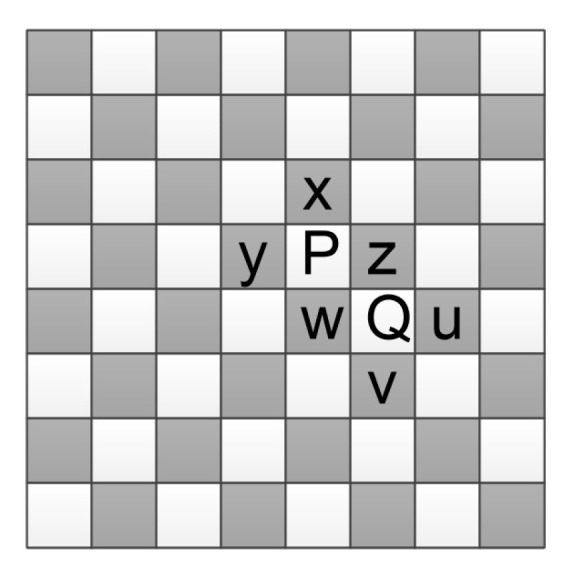
The chessboard binary-map, where pixels are divided to two disjoint sets: a set of pixels marked as white and a set of pixels marked as black.

The conventional methods often use a fixed binary-map, such as first-row-first-column [11], parity-column [16], chessboard [10], and block [8]. For example, Figure 2 shows the chessboard binary-map, where the pixels in the black region are kept unchanged to predict the pixels in the white region. Referring to Figure 2, one can mark the pixels in the black region as “0” and the rest as “1”. Since the subsequent procedure relies heavily on the binary-map, different binary-maps result in different payload-distortion performance. Regardless of the embedding performance, using a fixed binary-map may allow an unauthorized decoder to reconstruct the directly embedded data.

To this end, we propose to use a *key-controlled* binary-map. It implies that the binary-map will always change due to a key, ensuring that an unauthorized receiver cannot produce the correct binary-pattern. Obviously, the traditional method is a special case of the proposed *key-controlled* binary-map. One may use a content-adaptive operation to generate the binary-map. Namely, with an initialized binary-map, one may further optimize it for improving performance. Algorithm 1 provides the pseudocode. Actually, with an initialized binary-map, one may just randomly select more elements to produce a new binary-map, which also ensures the system security.
**Algorithm 1** Binary-map generation procedure**Input:** A cover and a secret key.**Output:** A binary-map and the side information (if any).  1: Initialize a binary-map  2: **while** need optimization **do**  3: Optimize the binary-map  4: **end while**  5: **return** final binary-map and side information (if any)

### 3.2. Content Prediction

With a binary-map, the elements marked as “0” are used to predict those marked as “1”. The elements to be embedded may be *randomly* or *content-adaptively* distributed in the binary-map. We cannot directly use a fixed predictor depending on fixed neighbors. We propose to use a *dynamic* predictor for prediction. It means that a cover element is predicted from an indefinite number of its neighbors, e.g., a pixel is predicted from its four neighbors, and another one is predicted with its eight neighbors. There are two advantages: the security can be ensured, and the prediction accuracy may be improved.

For the latter, we take Figure 2 for explanation. If P has been predicted from {x, y, z, w}, instead of {z, w, u, v}, Q could be predicted from {P, z, w, u, v}. It is seen that the traditional predictors could be considered as a special dynamic predictor. Accordingly, a most important problem for content prediction is to design the dynamic predictor. It is necessary to find an efficient method to orderly predict the cover elements. One may predict the cover elements by a row-by-row manner, which, however, has limited generality. We propose a method called *degree-first prediction (DFP)*, which consists of *element-wise selection* and *element-wise prediction*, to orderly predict the elements. As shown in Algorithm 2, in each time, the first step selects an element out according to its *degree*. The second step determines its prediction value with a dynamic predictor. The degree of an element is a scalar that denotes the prediction priority. A larger degree means a higher priority. The degree of a cover element relies on its local context.
**Algorithm 2** Degree-first prediction (DFP) procedure**Input:** A cover, a binary-map and a secret key.
**Output:** A prediction version of the cover.  1: Initialization  2: **while** exist an element to be processed **do**  3:  Choose an unprocessed element that has a largest degree  4:  Find the prediction value (with a dynamic predictor)  5:  Record the prediction value  6:  Mark the element as processed  7:  Update the degrees of the rest elements to be processed  8: **end while**  9: **return** the prediction version of the cover

### 3.3. Content Selection

The content selection aims to identify the embedding order. In image-based algorithms [10,17], the content selection is also named as pixel selection or sorting. For content selection, one has to define a local-complexity function to evaluate the prediction accuracy. Usually, a smaller local-complexity value implies better prediction accuracy. Thus, the cover elements can be orderly collected by sorting their local-complexities and then be orderly embedded. Algorithm 3 shows the pseudocode. In some methods, e.g., in Reference [17], a threshold, rather than sorting, may be used to take advantage of the smooth elements as much as possible. And these methods are in a sense the same as the method using sorting.
**Algorithm 3** Local-complexity-based selection procedure**Input:** A cover, a binary-map, the corresponding prediction version of the cover and the secret key.
**Output:** An ordered element sequence.  1: Initialization (e.g., empty the sequence)  2: **while** exist an element to be processed **do**  3:  Find/update all required local-complexity values  4:  Select an element with a smallest complexity value  5:  Append the element to the sequence  6:  Mark the element as processed  7:** end while**  8: **return** the ordered sequence

### 3.4. Data Embedding

After content selection, the data hider would perform data embedding. It is desirable to use HS or its variants to embed secret data since they can lead to superior payload-distortion performance. In detail, after generating an ordered pixel sequence, the data hider can determine out the corresponding PE sequence (PES). Thereafter, by using suitable histogram bin-pairs, the secret data can be easily embedded into the corresponding PEH. The traditional methods often empirically tune the shifting parameters, which is not desirable in applications. For a *single-layer* embedding, one may easily reproduce the reported simulation results. However, when adopting *multi-layer* embedding, it is actually time consuming and makes it hard for a reader to do the simulation due to the large space for searching suitable embedding parameters. To deal with this problem, in this paper, we propose a fast histogram shifting optimization (FastHiSO) algorithm to find *near-optimal* parameters. There are three advantages for the FastHiSO comparing with traditional operations: (1) the time complexity is relatively low; (2) a better payload-distortion performance can be achieved; and (3) the FastHiSO is deterministic, rather than empirical. In the following, we use digital image as the cover for detailing the FastHiSO algorithm.

Mathematically, we define $x^{\left(t\right)}\left(t\geq 0\right)$ as the cover image after embedded with *t* times. For simplicity, let $x^{\left(t\right)}=\left({x_{1}}^{\left(t\right)},{x_{2}}^{\left(t\right)},\ldots ,{x_{n}}^{\left(t\right)}\right)\in X={\left\{I\right\}}^{n}$ be an *n*-pixel cover image with the pixel range I, e.g., I={0,1,…,255} for 8-bit grayscale images. For a given message, we use $x^{\left(0\right)}$ and $x^{\left(t\right)}$ to generate the marked image $x^{\left(t+1\right)}\left(t\geq 0\right)$ by HS. Our goal is to find such HS parameters that both the distortion *D* between $x^{\left(0\right)}$ and $x^{\left(t+1\right)}$ and the computational cost can be kept low at the same time. We limit ourselves to a commonly used *additive-distortion* measure, i.e., mean squared error (MSE):

(1) $$D\left(x^{\left(0\right)},x^{\left(t+1\right)}\right)=\frac{1}{n}\cdot \sum_{i=1}^{n}{\left({x_{i}}^{\left(0\right)}-{x_{i}}^{\left(t+1\right)}\right)}^{2}.$$

Let h(v) represent the frequency of the PEH bin with a value of *v*, where −|I|<v<|I|. To embed data, with the generated PEH, we shift some PEs to vacate empty positions, and then embed secret bits by shifting the peak bins to the empty positions. Let $c^{\left(t\right)}=\left({c_{1}}^{\left(t\right)},{c_{2}}^{\left(t\right)},\ldots ,{c_{n_{t}}}^{\left(t\right)}\right),n_{t}\leq n$, be all the pixels to be embedded. We, respectively, denote the prediction of $c^{\left(t\right)}$ and its marked version by ${\hat{c}}^{\left(t\right)}=\left({{\hat{c}}_{1}}^{\left(t\right)},{{\hat{c}}_{2}}^{\left(t\right)},\ldots ,{{\hat{c}}_{n_{t}}}^{\left(t\right)}\right)$ and $s^{\left(t\right)}=\left({s_{1}}^{\left(t\right)},{s_{2}}^{\left(t\right)},\ldots ,{s_{n_{t}}}^{\left(t\right)}\right)$. Thus, we can determine the PEs $e^{\left(t\right)}=\left({e_{1}}^{\left(t\right)},{e_{2}}^{\left(t\right)},\ldots ,{e_{n_{t}}}^{\left(t\right)}\right)$ as follows:

(2) $${e_{i}}^{\left(t\right)}={c_{i}}^{\left(t\right)}-{{\hat{c}}_{i}}^{\left(t\right)},\left(1\leq i\leq n_{t}\right).$$

For data embedding, we first select two peak points $\left({l_{p}}^{\left(t\right)},{r_{p}}^{\left(t\right)}\right)$ and two integers $\left({T_{l}}^{\left(t\right)},{T_{r}}^{\left(t\right)}\right)$, where ${l_{p}}^{\left(t\right)}<{r_{p}}^{\left(t\right)}$, ${T_{l}}^{\left(t\right)}>0$, ${T_{r}}^{\left(t\right)}>0$. Then, secret bits can be embedded by using the HS operation, namely

(3) $${s_{i}}^{\left(t\right)}={{\hat{c}}_{i}}^{\left(t\right)}+{{\hat{e}}_{i}}^{\left(t\right)}={{\hat{c}}_{i}}^{\left(t\right)}+{e_{i}}^{\left(t\right)}+\Delta \left({e_{i}}^{\left(t\right)}\right),$$

where

(4) $$\Delta \left({e_{i}}^{\left(t\right)}\right)=\left\{\begin{array}{cc} -{T_{l}}^{\left(t\right)}, & \;\mathrm{if}\;{e_{i}}^{\left(t\right)}\leq {l_{p}}^{\left(t\right)}-{T_{l}}^{\left(t\right)};\\ {T_{r}}^{\left(t\right)}, & \;\mathrm{if}\;{e_{i}}^{\left(t\right)}\geq {r_{p}}^{\left(t\right)}+{T_{r}}^{\left(t\right)};\\ {e_{i}}^{\left(t\right)}-{r_{p}}^{\left(t\right)}+b, & \;\mathrm{else}\;\mathrm{if}\;{e_{i}}^{\left(t\right)}\geq {r_{p}}^{\left(t\right)};\\ {e_{i}}^{\left(t\right)}-{l_{p}}^{\left(t\right)}-b, & \;\mathrm{else}\;\mathrm{if}\;{e_{i}}^{\left(t\right)}\leq {l_{p}}^{\left(t\right)};\\ 0, & \;\mathrm{otherwise}.\; \end{array}\right.$$

Here, b∈{0,1} is the current bit to be embedded.

We use $o^{\left(t\right)}=\left({o_{1}}^{\left(t\right)},{o_{2}}^{\left(t\right)},\ldots ,{o_{n_{t}}}^{\left(t\right)}\right)$ to denote the original pixel values of $c^{\left(t\right)}$ in $x^{\left(0\right)}$. For the pixels not belonging to $c^{\left(t\right)}$, the introduced distortion can be roughly considered as fixed since we will not embed secret data into these pixels (though we may alter some pixels prior to embedding, e.g., we may empty some LSBs to store the secret key). Therefore, for (t+1)-layer (t≥0) embedding (i.e., to generate $x^{\left(t+1\right)}$), our optimization task is

(5) $$D\left(x^{\left(0\right)},x^{\left(t+1\right)}\right)=\min_{{l_{p}}^{\left(t\right)},{r_{p}}^{\left(t\right)},{T_{l}}^{\left(t\right)},{T_{r}}^{\left(t\right)}}\;\;\frac{1}{n}\cdot \sum_{i=1}^{n_{t}}{\left({s_{i}}^{\left(t\right)}-{o_{i}}^{\left(t\right)}\right)}^{2}+\frac{1}{n}\cdot C,$$

where *C* is a constant. We further have

(6) $$\begin{array}{cc} \sum_{i=1}^{n_{t}}{\left({s_{i}}^{\left(t\right)}-{o_{i}}^{\left(t\right)}\right)}^{2} & =\sum_{i=1}^{n_{t}}{\left({{\hat{c}}_{i}}^{\left(t\right)}+{{\hat{e}}_{i}}^{\left(t\right)}-{o_{i}}^{\left(t\right)}\right)}^{2}\\ \; & =\sum_{i=1}^{n_{t}}{\left({c_{i}}^{\left(t\right)}-{o_{i}}^{\left(t\right)}+{{\hat{e}}_{i}}^{\left(t\right)}-{e_{i}}^{\left(t\right)}\right)}^{2}\\ \; & =\sum_{i=1}^{n_{t}}{\left({\alpha_{i}}^{\left(t\right)}+{\beta_{i}}^{\left(t\right)}\right)}^{2}, \end{array}$$

where ${\alpha_{i}}^{\left(t\right)}={c_{i}}^{\left(t\right)}-{o_{i}}^{\left(t\right)}$ and ${\beta_{i}}^{\left(t\right)}={{\hat{e}}_{i}}^{\left(t\right)}-{e_{i}}^{\left(t\right)}=\Delta \left({e_{i}}^{\left(t\right)}\right)$.

Therefore, it is inferred that our final optimization task is

(7) $$J\left(x^{\left(0\right)},x^{\left(t+1\right)}\right)=\min_{{l_{p}}^{\left(t\right)},{r_{p}}^{\left(t\right)},{T_{l}}^{\left(t\right)},{T_{r}}^{\left(t\right)}}\;\;\sum_{i=1}^{n_{t}}{\left({\alpha_{i}}^{\left(t\right)}+{\beta_{i}}^{\left(t\right)}\right)}^{2}.$$

Obviously, all ${\alpha_{i}}^{\left(t\right)}\;\left(1\leq i\leq n_{t}\right)$ are constants before embedding, meaning that they can be determined in advance. It can be seen that $|{\beta_{i}}^{\left(t\right)}|=|\Delta \left({e_{i}}^{\left(t\right)}\right)|\leq \max\left\{{T_{l}}^{\left(t\right)},{T_{r}}^{\left(t\right)}\right\}$ for all $1\leq i\leq n_{t}$, which gives us the chance to quickly determine $J(x^{\left(0\right)},x^{\left(t+1\right)})$ for fixed $c^{\left(t\right)}$, $o^{\left(t\right)}$ and $e^{\left(t\right)}$. In detail, we use a 2D histogram-matrix $H=\{H_{u,v}|-|I|\leq u,v\leq |I|\}$ to record the occurrence of every possible integer-pair (u,v) in advance, where *u* represents the possible value of ${\alpha_{i}}^{\left(t\right)}$, and *v* shows the possible value of ${e_{i}}^{\left(t\right)}$. In this way, Equation (7) is equivalent to

(8) $$J\left(x^{\left(0\right)},x^{\left(t+1\right)}\right)=\min_{{l_{p}}^{\left(t\right)},{r_{p}}^{\left(t\right)},{T_{l}}^{\left(t\right)},{T_{r}}^{\left(t\right)}}\;\;\sum_{u,v}H_{u,v}\cdot {\left(u+\Delta \left(v\right)\right)}^{2}.$$

According to Equation (4), we further have

(9) $$\begin{array}{cc} \; & \sum_{u,v}H_{u,v}\cdot {\left(u+\Delta \left(v\right)\right)}^{2}=\sum_{u}\sum_{v}H_{u,v}\cdot {\left(u+\Delta \left(v\right)\right)}^{2}=\sum_{u}\sum_{v\leq {l_{p}}^{\left(t\right)}-{T_{l}}^{\left(t\right)}}H_{u,v}\cdot {\left(u-T_{l}\right)}^{2}\\ \; & \;+\sum_{u}\sum_{v\geq {r_{p}}^{\left(t\right)}+{T_{r}}^{\left(t\right)}}H_{u,v}\cdot {\left(u+T_{r}\right)}^{2}+\sum_{u}\sum_{{l_{p}}^{\left(t\right)}-{T_{l}}^{\left(t\right)}+1\leq v\leq {l_{p}}^{\left(t\right)}}H_{u,v}\cdot {\left(u+v-{l_{p}}^{\left(t\right)}-b\right)}^{2}\\ \; & \;+\sum_{u}\sum_{{r_{p}}^{\left(t\right)}\leq v\leq {r_{p}}^{\left(t\right)}+T_{r}-1}H_{u,v}\cdot {\left(u+v-{r_{p}}^{\left(t\right)}+b\right)}^{2}+\sum_{u}\sum_{{l_{p}}^{\left(t\right)}+1\leq v\leq {r_{p}}^{\left(t\right)}-1}H_{u,v}\cdot u^{2} \end{array},$$

where *b* is the corresponding bit to be embedded.

Since the embedded bits are always encrypted, to keep the computational cost low, we will consider

(10) $$\begin{array}{cc} \; & \sum_{u}\sum_{{l_{p}}^{\left(t\right)}-{T_{l}}^{\left(t\right)}+1\leq v\leq {l_{p}}^{\left(t\right)}}H_{u,v}\cdot {\left(u+v-{l_{p}}^{\left(t\right)}-b\right)}^{2}\approx \\ \; & \;\;\;\;\;\;\;\;\;\;\;\;\;\;\;\;\;\;\sum_{u}\sum_{{l_{p}}^{\left(t\right)}-{T_{l}}^{\left(t\right)}+1\leq v\leq {l_{p}}^{\left(t\right)}}\frac{H_{u,v}}{2}\cdot {\left(u+v-{l_{p}}^{\left(t\right)}\right)}^{2}\\ \; & \;\;\;\;\;\;\;\;\;\;\;\;\;\;\;\;\;\;\;\;\;\;\;\;\;\;\;\;\;\;\;\;\;\;\;\;+\sum_{u}\sum_{{l_{p}}^{\left(t\right)}-{T_{l}}^{\left(t\right)}+1\leq v\leq {l_{p}}^{\left(t\right)}}\frac{H_{u,v}}{2}\cdot {\left(u+v-{l_{p}}^{\left(t\right)}-1\right)}^{2}, \end{array}$$

and

(11) $$\begin{array}{cc} \; & \sum_{u}\sum_{{r_{p}}^{\left(t\right)}\leq v\leq {r_{p}}^{\left(t\right)}+T_{r}-1}H_{u,v}\cdot {\left(u+v-{r_{p}}^{\left(t\right)}+b\right)}^{2}\approx \\ \; & \;\;\;\;\;\;\;\;\;\;\;\;\;\;\;\;\;\;\sum_{u}\sum_{{r_{p}}^{\left(t\right)}\leq v\leq {r_{p}}^{\left(t\right)}+T_{r}-1}\frac{H_{u,v}}{2}\cdot {\left(u+v-{r_{p}}^{\left(t\right)}\right)}^{2}\\ \; & \;\;\;\;\;\;\;\;\;\;\;\;\;\;\;\;\;\;\;\;\;\;\;\;\;\;\;\;\;\;\;\;\;\;\;\;+\sum_{u}\sum_{{r_{p}}^{\left(t\right)}\leq v\leq {r_{p}}^{\left(t\right)}+T_{r}-1}\frac{H_{u,v}}{2}\cdot {\left(u+v-{r_{p}}^{\left(t\right)}+1\right)}^{2}. \end{array}$$

Algorithm 4 shows the FastHiSO procedure. In Algorithm 4, the 2D histogram-matrix H can be determined with a time complexity of $O(n_{t})$, which is linear with respect to the number of cover elements to be embedded. For Line 6 in Algorithm 4, according to Equation (9), the time complexity is $O(|I{|}^{2})$ at the worst case for the fixed $\left({l_{p}}^{\left(t\right)},{r_{p}}^{\left(t\right)},{T_{l}}^{\left(t\right)},{T_{r}}^{\left(t\right)}\right)$. Since the PEH is Gaussian-like [17], the optimal values of ${l_{p}}^{\left(t\right)}$ and ${r_{p}}^{\left(t\right)}$ should be close to zero. It indicates that, from the empirical point of view, the absolute values of ${l_{p}}^{\left(t\right)}$ and ${r_{p}}^{\left(t\right)}$ can be limited to a small range, e.g., $\max\{|{l_{p}}^{\left(t\right)}|,|{r_{p}}^{\left(t\right)}|\}\leq 64$. The values of ${T_{l}}^{\left(t\right)}$ and ${T_{r}}^{\left(t\right)}$ can be also limited to a small range, as well, e.g., $\max\{{T_{l}}^{\left(t\right)},{T_{r}}^{\left(t\right)}\}\leq 4$. In this way, the near-optimal parameters can be quickly determined, and the corresponding complexity can be approximately expressed as $O(kn_{t})$, where $k<<n_{t}$ is a relatively small integer.

With the proposed FastHiSO algorithm, one can easily embed secret data into the cover pixel-sequence. The embedding process is described in Algorithm 5. To avoid the underflow/overflow problem, we need to adjust the pixels with boundary values into the reliable range in advance. The preprocessed pixels should be recorded as side information, which will be self-embedded.
**Algorithm 4** Fast histogram shifting optimization (FastHiSO)**Input:** The ordered cover sequence $c^{\left(t\right)}$, the original sequence $o^{\left(t\right)}$, the prediction sequence ${\hat{c}}^{\left(t\right)}$, and the payload size ρ.**Output:** 
$\left({l_{p}}^{\left(t\right)},{r_{p}}^{\left(t\right)},{T_{l}}^{\left(t\right)},{T_{r}}^{\left(t\right)}\right)$  1: Empty H
  2: **for** 
$1\leq i\leq n_{t}$ **do**  3: Find $u={\alpha_{i}}^{\left(t\right)}$ and $v={e_{i}}^{\left(t\right)}$  4: Set $H_{u,v}\leftarrow H_{u,v}+1$  5: **end for**  6: Find the near-optimal $\left({l_{p}}^{\left(t\right)},{r_{p}}^{\left(t\right)},{T_{l}}^{\left(t\right)},{T_{r}}^{\left(t\right)}\right)$ with Equations (8)–(11), subject to the payload size ρ.  7: **return**
$\left({l_{p}}^{\left(t\right)},{r_{p}}^{\left(t\right)},{T_{l}}^{\left(t\right)},{T_{r}}^{\left(t\right)}\right)$


**Algorithm 5** Data embedding procedure
**Input:** The ordered cover sequence $c^{\left(t\right)}$, the original sequence $o^{\left(t\right)}$, the prediction sequence ${\hat{c}}^{\left(t\right)}$, the required payload L, and the secret key $k_{\mathrm{emb}}$.**Output:** The marked pixel-sequence $s^{\left(t\right)}$.  1: Call FastHiSO to find near-optimal HS parameters  2: Embed L into the corresponding PEH  3: Construct the marked pixel-sequence $s^{\left(t\right)}$  4: **return** $s^{\left(t\right)}$ {thereafter, we further generate $x^{\left(t+1\right)}$}


### 3.5. Data Extraction and Cover Recovery

Once secret data is successfully embedded, the resulting marked object will be sent to the desired receiver, who should be able to reconstruct the original cover and extract the embedded data without error according to the secret key. The data extraction and cover recovery procedure can be considered as an inverse process to the data hider.

### 3.6. Relationships between Different Parts

There may exist interactions between the different parts. We present two different relationships for better generalization.

#### 3.6.1. Relationship between Binary-Map Generation, Content Prediction, and Content Selection

With an initialized binary-map, we can optimize it for realizing better payload-distortion performance. We may use the procedure similar to content prediction and selection, e.g., Algorithm 6 shows an example of optimizing a binary-map using Algorithms 2 and 3.
**Algorithm 6** Binary-map generation using Algorithms 2 and 3**Input:** A cover and a secret key.**Output:** A binary-map.  1: Initialize a binary-map  2: **while** need optimization **do**  3: Call Algorithm 2 to predict the cover  4: Call Algorithm 3 to generate a sequence  5: Choose a certain number of elements (which are marked as “1”) out from the ordered sequence  6: Mark them as ”0” and update the binary-map  7: **end while**  8: **return** the final binary-map

#### 3.6.2. Relationship between Content Prediction and Content Selection

For content prediction, the data hider has to identify the prediction order. As mentioned above, each time, the data hider chooses an element with a largest degree for prediction. For content selection, the data hider uses a local-complexity function to choose an element with the lowest complexity in each time. It indicates that every to-be-processed element will be associated with a local-complexity value. As the degree and local-complexity are scalars, they may affect each other. e.g., in Algorithm 2, after executing Line 7, one can directly append the processed element to a sequence. Thus, an ordered element sequence presented in Algorithm 3 can be also generated after content prediction.

### 3.7. Other Perspectives

Next, two different types of dynamic predictors, i.e., raw-content-independent (RCI)-based predictor and raw-content-dependent (RCD)-based predictor are considered. RCI-based predictor uses the marked or predicted values of the neighbors of the present element as the prediction context. It means that the prediction process for an element may not affect the prediction process of another one directly. Thus, the content prediction order for a data receiver is probably identical to the sender. In contrast, RCD-based predictor uses the raw content, which can benefit prediction accuracy. Thus, we should ensure that the raw values of the context have been obtained before prediction.

Intuitively, a dynamic predictor enables us to predict different cover elements from different contexts. Actually, a general dynamic predictor also implies that:

- *Fusion of multiple subpredictors*: The conventional methods use a single predictor. Actually, they can be treated as a fusion of multiple subpredictors. We take median edge detector (MED) [13] for explanation, i.e.,
  (12) $$\begin{array}{c} \hat{x}=\left\{\begin{array}{cc} \min\{v_{1},v_{3}\}, & \mathrm{if}\;v_{4}\geq \max\left\{v_{1},v_{3}\right\},\\ \max\{v_{1},v_{3}\}, & \mathrm{if}\;v_{4}\leq \min\left\{v_{1},v_{3}\right\},\\ v_{1}+v_{3}-v_{4}, & \mathrm{otherwise}, \end{array}\right. \end{array}$$
  where $v_{1}$, $v_{3}$, and $v_{4}$ are specific neighbors of *x*. It can be seen that the MED essentially uses three subpredictors, i.e., $\min\{v_{1},v_{3}\}$, $\max\{v_{1},v_{3}\}$, and $v_{1}+v_{3}-v_{4}$. Therefore, it is inferred that a dynamic predictor corresponds to a fusion of multiple subpredictors. A key work is to choose the suitable subpredictor according to the local context.
- *Fusion of multiple subhistograms*: The histogram to be embedded also can be regarded as a fusion of multiple subhistograms as a subpredictor corresponds to a subhistogram. Though we may not directly use the subhistograms separately, it inspires us to divide a histogram into multiple subhistograms for payload-distortion optimization, which has been exploited by Li et al. [18].
- *Fusion of multiple subcovers*: Different subhistograms are corresponding to different subcovers even though the elements belonging to a subcover may be widely or near-randomly distributed in the original cover. In other words, we may divide the cover into subcovers for payload-distortion optimization since different subcovers may have different texture characteristics. For example, a cover image may be divided into disjoint blocks. Notice that this perspective no longer focuses on only the dynamic predictor, but rather the design of an RDH system, e.g., as in Reference [19].

In addition, by default, we consider the cover element to be embedded as a single value for better understanding. Actually, the “element” can be a vector. For example, in Reference [20], two pixels are grouped as a pair to carry the secret data.

## 4. Two Examples Based on Proposed Framework

In this section, we will present two novel RDH algorithms based on the proposed framework to show the efficiency and applicability of the proposed framework.

### 4.1. Prediction-Error of Prediction Error (PPE)-Based RDH

Due to the spatial correlations between neighboring pixels, many existing works use PEs to carry the secret data. Actually, there also exist correlations between neighboring PEs. An evidence can be found in the prediction mechanism of video lossy compression. For example, in intra prediction, the prediction block for an intra 4×4 luma macroblock is generated with 9 possible prediction modes. Then, to improve the coding efficiency, the prediction mode of a luma macroblock is predicted from the prediction modes of neighboring luma macroblocks since correlations also exist between the neighboring prediction modes. The success of steganalysis by modeling the differences between neighboring pixels with low-order Markov chains [21] also reveals that correlations exist between the neighboring PEs if we consider the differences as a kind of PEs. Based on this perspective, we here present a prediction-error of prediction error (PPE)-based RDH algorithm, which is an extension of Reference [14].

With a cover image **x** $\in {\left\{0,1,\ldots ,2^{d}-1\right\}}^{n_{1}\times n_{2}}$ and a binary-map $b_{\mathrm{map}}\in {\left\{0,1\right\}}^{n_{1}\times n_{2}}$, we define a neighbor-set $D_{0}\left(x_{i,j}\right)$ for each $x_{i,j}\in x$ corresponding to $b_{i,j}=1\;\left(\in b_{\mathrm{map}}\right)$. $D_{0}\left(x_{i,j}\right)$ includes the neighboring pixels of $x_{i,j}$ that are marked as “0” in $b_{\mathrm{map}}$. We first use the pixels in $D_{0}\left(x_{i,j}\right)$ to predict $x_{i,j}$, i.e., ${\hat{x}}_{i,j}=f_{0}\left(D_{0}\left(x_{i,j}\right)\right)$, from which we can obtain the PE, denoted by $e_{i,j}=x_{i,j}-{\hat{x}}_{i,j}$. Then, $e_{i,j}$ is further predicted by exploiting the PEs of the pixels in $D_{0}\left(x_{i,j}\right)\cup D_{1}\left(x_{i,j}\right)$, i.e., ${\hat{e}}_{i,j}=f_{1}\left(D_{0}\left(x_{i,j}\right)\cup D_{1}\left(x_{i,j}\right)\right)$. Here, $D_{1}\left(x_{i,j}\right)$ represents the pixels adjacent to at least one pixel in $D_{0}\left(x_{i,j}\right)$. Note that the definition of $D_{0}\left(x_{i,j}\right)$ and $D_{1}\left(x_{i,j}\right)$ may be different from each other. The PPE of $x_{i,j}$ is described as:

(13) $${e^{\circ }}_{i,j}=e_{i,j}-{\hat{e}}_{i,j}=x_{i,j}-f_{0}\left(D_{0}\left(x_{i,j}\right)\right)-f_{1}\left(D_{0}\left(x_{i,j}\right)\cup D_{1}\left(x_{i,j}\right)\right).$$

**Figure 3:**
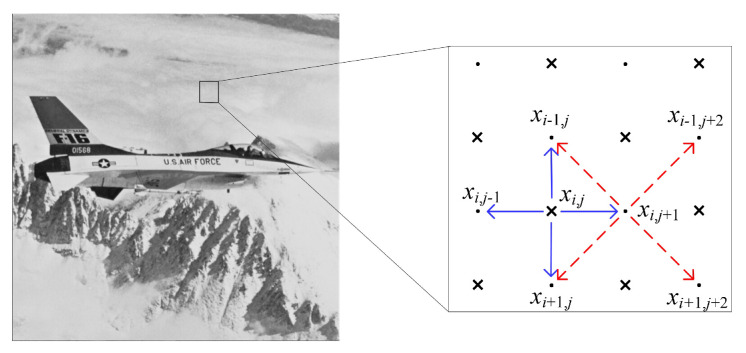
The pixel prediction pattern. $x_{i,j}$ is predicted from four neighbors in the dot set, and $x_{i,j+1}$ is predicted from four neighbors still in the dot set.

We use the chessboard pattern to construct $b_{\mathrm{map}}$. As shown in Figure 3, $D_{0}\left(x_{i,j}\right)=\{x_{i-1,j},x_{i,j+1}$, $x_{i+1,j},x_{i,j-1}\}$ is first utilized to predict $x_{i,j}$. Then, the PEs of the pixels in $\{x_{i-1,j},x_{i,j+1}$, $x_{i+1,j},x_{i,j-1}\}$ are used to predict the PE of $x_{i,j}$. The prediction process of neighboring pixels is different from $x_{i,j}$, e.g., $x_{i,j+1}$ is predicted from $\{x_{i-1,j},x_{i-1,j+2}$, $x_{i+1,j},x_{i+1,j+2}\}$ since the cross set is to be embedded and the dot set for unchanged.

As shown in Figure 3, we first predict $x_{i,j}$ along the horizontal and vertical directions. The two directional predictors are:

(14) $${x_{i,j}}^{{}^{\prime }}=\frac{x_{i,j-1}+x_{i,j+1}}{2},\;{x_{i,j}}^{{}^{\prime \prime }}=\frac{x_{i-1,j}+x_{i+1,j}}{2}.$$

Then, ${\hat{x}}_{i,j}$ is determined by:

(15) $${\hat{x}}_{i,j}=\mathrm{Round}\left(w_{i,j}\cdot {x_{i,j}}^{{}^{\prime }}+\left(1-w_{i,j}\right)\cdot {x_{i,j}}^{{}^{\prime \prime }}\right).$$

Here, Round(·) returns the nearest integer, $w_{i,j}$ is defined as:

(16) $$w_{i,j}=\frac{{\sigma_{i,j}}^{{}^{\prime \prime }}}{{\sigma_{i,j}}^{{}^{\prime }}+{\sigma_{i,j}}^{{}^{\prime \prime }}},$$

where

(17) $${\sigma_{i,j}}^{{}^{\prime }}=\frac{1}{3}\cdot \sum_{v\in \{x_{i,j-1},{x_{i,j}}^{{}^{\prime }},x_{i,j+1}\}}{\left(v-\frac{{x_{i,j}}^{{}^{\prime }}+{x_{i,j}}^{{}^{\prime \prime }}}{2}\right)}^{2}.$$

(18) $${\sigma_{i,j}}^{{}^{\prime \prime }}=\frac{1}{3}\cdot \sum_{v\in \{x_{i-1,j},{x_{i,j}}^{{}^{\prime \prime }},x_{i+1,j}\}}{\left(v-\frac{{x_{i,j}}^{{}^{\prime }}+{x_{i,j}}^{{}^{\prime \prime }}}{2}\right)}^{2}.$$

It is straightforward to process other pixels in the cross set and the dot set with the similar procedure, e.g., $x_{i,j+1}$ will be predicted from the two diagonal directions. Thereafter, we use the average value of the PEs of pixels in $D_{0}\left(x_{i,j}\right)$ as the prediction of $e_{i,j}$, i.e., ${\hat{e}}_{i,j}$. And, the corresponding PPE can be determined according to Equation (13). Therefore, all PPEs of pixels in the cross set can be determined. By defining a local-complexity function, the PPEs can be sorted in a decreasing order of the prediction accuracy, which can benefit embedding performance. Here, the local-complexity function is defined as:

(19) $$\epsilon_{i,j}={\left[\frac{1}{6}\cdot \sum_{s=1}^{6}{\left({\varrho_{i,j}}^{\left(s\right)}-\sum_{t=1}^{6}{\varrho_{i,j}}^{\left(t\right)}/6\right)}^{2}\right]}^{1/2},$$

where ${\varrho_{i,j}}^{\left(1\right)}=\left|x_{i-1,j}-x_{i,j+1}\right|$, ${\varrho_{i,j}}^{\left(2\right)}=\left|x_{i-1,j}-x_{i+1,j}\right|$, ${\varrho_{i,j}}^{\left(3\right)}=\left|x_{i-1,j}-x_{i,j-1}\right|$, ${\varrho_{i,j}}^{\left(4\right)}=\left|x_{i,j+1}-x_{i+1,j}\right|$, ${\varrho_{i,j}}^{\left(5\right)}=\left|x_{i,j+1}-x_{i,j-1}\right|$, and ${\varrho_{i,j}}^{\left(6\right)}=\left|x_{i+1,j}-x_{i,j-1}\right|$. Accordingly, an ordered PPE sequence can be generated. We sincerely refer the readers to References [14,22] for more details. Note that one may use other efficient binary-maps, pixel prediction procedures, and local-complexity functions. Finally, for a payload, by applying the proposed FastHiSO algorithm, one can quickly find the near-optimal parameters and embed the secret bits into the PPE histogram according to the operation similar to Equations (3) and (4). For a receiver, data extraction and image recovery correspond to a reverse operation.

**Figure 4:**
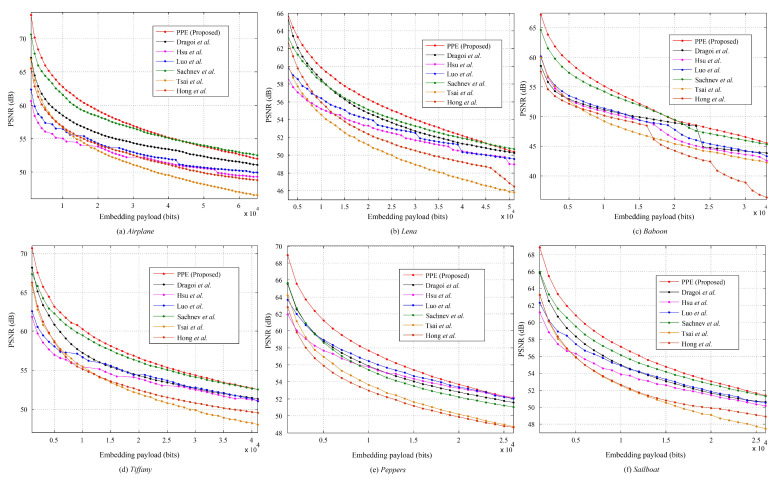
The payload-distortion performance comparison between the state-of-the-art methods of Tsai et al. [8], Sachnev et al. [10], Hong et al. [11], Luo et al. [12], Hsu et al. [23], Dragoi et al. [24], and the proposed method (PPE).

We present some experimental results to show the efficiency of PPE-based RDH method. Six standard test images, from smooth to complex (http://sipi.usc.edu/database/, accessed on 10 January 2021): *Airplane*, *Lena*, *Baboon*, *Tiffany*, *Peppers*, and *Sailboat* (i.e., *Fishing boat*); all are grayscaled with a size of 512×512 used. During data embedding, we set $\max\{|{l_{p}}^{\left(t\right)}|,|{r_{p}}^{\left(t\right)}|\}\leq 255$ and $\max\{{T_{l}}^{\left(t\right)},{T_{r}}^{\left(t\right)}\}\leq 1$. Figure 4 shows the payload-distortion performance comparison between some prediction-based RDH works and the proposed method. It is seen that, for relatively low embedding rates, the proposed method significantly outperforms the related works, meaning that the data embedding performance can benefit from the cover PPEs and the FastHiSO algorithm. On the other hand, in Figure 4, when the embedding payload increases, the PSNR improvement is not significant, even slightly bad (e.g., *Airplane*). It means that the generated PPE histogram (PPEH) has its limitation. Overall, the proposed method can still provide better payload-distortion performance compared to a part of related works and maintain satisfactory trade-off between the embedding payload and the distortion.

In this subsection, we use the PPEs to carry a payload. It is straightforward to apply higher-order PEs to hide the secret data. It can be inferred that both the PPEs and the higher-order PEs essentially correspond to a kind of *calibration* operation to improve the prediction accuracy so as to provide better performance. Any predictor can be written as a form of predicting a pixel itself, so that PE-based RDH and PPE-based RDH, as well as the higher-order PE-based RDH, are all generalized by the proposed framework.

Therefore, a core work for prediction-based RDH system is to keep the *calibration* operation as accurate as possible. In this paper, we will not study the prediction accuracy in depth since it is not the main interest of this paper. The traditional methods usually have no *calibration* term (or are considered as constant).

### 4.2. Dynamic Selection-and-Prediction (DSP)-Based RDH

The aforementioned algorithm uses a fixed pattern to construct $b_{\mathrm{map}}$, providing better embedding performance compared to the related works. According to Kerckhoffs’s principle, this previously specified pattern may allow one to successfully reconstruct the marked histogram, and extract the directly embedded information, which is not desirable for applications. To overcome this drawback, we here use a key to initialize the binary-map generation such that the final $b_{\mathrm{map}}$ is always changing due to the key.

For RDH, smooth regions often correspond to better payload-distortion performance. To improve the performance, we can optimize the initialized $b_{\mathrm{map}}$ to select smooth regions out for data embedding as long as the smooth pixels can carry the payload. However, since all “1”s in $b_{\mathrm{map}}$ will be randomly or content-adaptively distributed due to the key, we cannot directly use such predictors that rely on the specified neighbors. To deal with this problem, we propose to use a *dynamic* predictor for pixel prediction. That is why we call it as *dynamic selection-and-prediction (DSP)*-based RDH method. Thereafter, with a well-defined local-complexity function and the FastHiSO algorithm, the secret data can be embedded into the corresponding PEH. It can be seen that DSP-based RDH meets the requirement of our framework. In the following, we will describe the details of the proposed DSP-based RDH, which is an extension version of Reference [15].

For self-contained, let X be an image with n=h×w pixels; for compactness, we sometimes consider X as the set including all pixels and say “pixel $x_{i,j}$” meaning a pixel located at position (i,j), whose grayscale value is $x_{i,j}$. We first use a secret key to initialize $b_{\mathrm{map}}$, where “0”s are pseudo-randomly distributed. For simplicity, we use $S_{0}$ to denote the pixel-set containing all pixels marked as “0” in the initialized $b_{\mathrm{map}}$. Then, we optimize $b_{\mathrm{map}}$ by selecting more complex pixels out, denoted by $S_{1}$. Here, $S_{0}\cap S_{1}=\emptyset$ and $S_{0}\cup S_{1}\subset X$. Note that the pixels in $S_{0}\cup S_{1}$ will be marked as “0”, and that in $X\backslash \left(S_{0}\cup S_{1}\right)$ correspond to “1”. The generation of $S_{1}$ involves two steps, namely the *degree-first prediction (DFP)* and the *complexity-first selection (CFS)*.

*DFP Procedure:* We collect all pixels in $S_{0}$, and only exploit these pixels to predict the pixels in $X\backslash S_{0}$. The pixels are orderly predicted according to the associated degrees. The *degree* of a pixel is defined as the size of its *degree-set*, which is a subset of its *neighbor-set*. The neighbor-set of a pixel $x_{i,j}$ is defined as:

(20) $$N_{i,j}=\left\{x_{i+u,j+v}|1\leq u^{2}+v^{2}\leq r^{2};u,v\in Z\right\}.$$

By default, $r=\sqrt{2}$. Thus, except for boundary positions, the neighbor-set of a pixel consists of eight pixels. The degree-set of $x_{i,j}$ is then determined by:

(21) $$D_{i,j}=N_{i,j}\cap \left(S_{0}\cup A\right),$$

where A represents the pixel-set consisting of the pixels that have been previously predicted. We always have $S_{0}\cap A=\emptyset$.

A pixel with a larger degree will be predicted prior to that with a smaller degree. The reason is, only pixels in the degree-set are utilized to predict a pixel. Thus, a pixel with a larger degree can be predicted from more pixels, meaning that the pixel can be well predicted as more context are provided. That is why we consider the prediction as “degree-first prediction”. In this paper, the prediction of $x_{i,j}$ is defined as:

(22) $${\hat{x}}_{i,j}=\frac{\sum_{x_{u,v}\in \left(N_{i,j}\cap A\right)}{\hat{x}}_{u,v}+\sum_{x_{u,v}\in \left(N_{i,j}\cap S_{0}\right)}x_{u,v}}{|D_{i,j}|}.$$

Based on the above description, we describe the proposed DFP procedure as follows.

**(Step 1)** Set A=∅. For all $x_{i,j}\in X\backslash S_{0}$, compute $D_{i,j}$ with Equation (21). Mark all $x_{i,j}\in X\backslash S_{0}$ as unprocessed.

**(Step 2)** Select such a *unprocessed* pixel $x_{i,j}\in X\backslash S_{0}$ that has the largest degree in $X\backslash \left(S_{0}\cup A\right)$. If there are multiple pixels that have the largest degree, choose one according to a key or a specified rule. Find ${\hat{x}}_{i,j}$ with Equation (22).

**(Step 3)** Mark $x_{i,j}$ as processed and update A as $A\cup \{x_{i,j}\}$, and further update $D_{i,j}$ with Equation (21).

**(Step 4)** Terminate the procedure if all pixels in $X\backslash S_{0}$ are processed; otherwise, go to **(Step 2)**.

*CFS Procedure:* After all required pixels are predicted, we are to select a part of the predicted pixels out to constitute $S_{1}$. It relies on the local complexities of the pixels. Here, we define the local complexity of a predicted pixel $x_{i,j}$ as:

(23) $$\rho_{i,j}=\frac{\sum_{x_{u,v}\in P_{i,j}}{\left(x_{u,v}-{\hat{x}}_{i,j}\right)}^{2}+\sum_{x_{u,v}\in Q_{i,j}}{\left({\hat{x}}_{u,v}-{\hat{x}}_{i,j}\right)}^{2}}{|N_{i,j}|},$$

where $P_{i,j}=S_{0}\cap N_{i,j}$, $Q_{i,j}=N_{i,j}\backslash S_{0}$.

A larger local complexity indicates that the pixel is likely to be located at a more complex region. Every predicted pixel is associated with its local complexity. We sort the pixels by their local complexities in an increasing order. In this way, the $|S_{1}|$ pixels with largest complexity-values are chosen to constitute $S_{1}$, and the selected pixels are likely located at relatively complex regions. Since we only use the original values of pixels in $S_{0}$, both the data hider and receiver should be able to construct the identical $S_{0}\cup S_{1}$ with the key. Thereafter, we use the pixels in $S_{0}\cup S_{1}$ to predict the pixels in $X\backslash \left(S_{0}\cup S_{1}\right)$ by applying the proposed DFP procedure. An ordered pixel-sequence together with the corresponding PEs can be generated according to the proposed CFS procedure. With the resulting PEH and the FastHiSO, the secret data can be sucessfully embedded.

**Figure 5:**
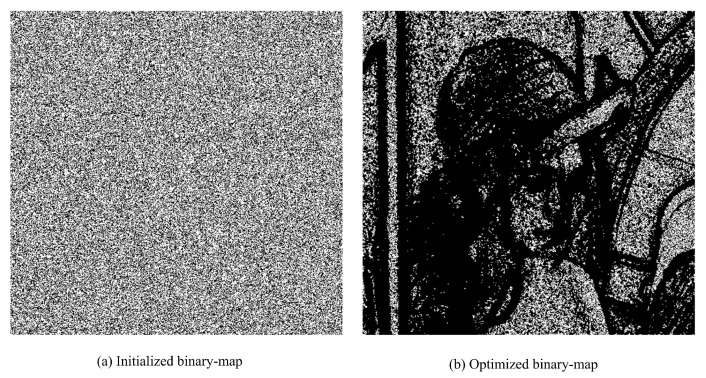
An example of the proposed binary-map generation procedure tested on *Lena*: $r=\frac{|S_{0}\cup S_{1}|}{\left|X\right|}\times 100\%=80\%$ (*black* regions in optimized version).

We present some experimental results to show the embedding performance. The system parameters mainly include $|S_{0}|$, $|S_{1}|$ and the secret key. For a payload, it is free to choose $|S_{0}|$ and $|S_{1}|$. For convenience, we consider $|S_{0}\left|=\right|S_{1}|$ in default, which may be not optimal. Figure 5 shows the distribution of $S_{0}$ and $S_{0}\cup S_{1}$ (black regions) tested on *Lena* image due to proposed binary-map generation procedure. It is seen that the proposed binary-map generation procedure can *auto-capture* the relatively smooth pixels (*white regions*) for data hiding, which is quite desirable for practice.

#### 4.2.1. Evaluation on Standard Images

**Figure 6:**
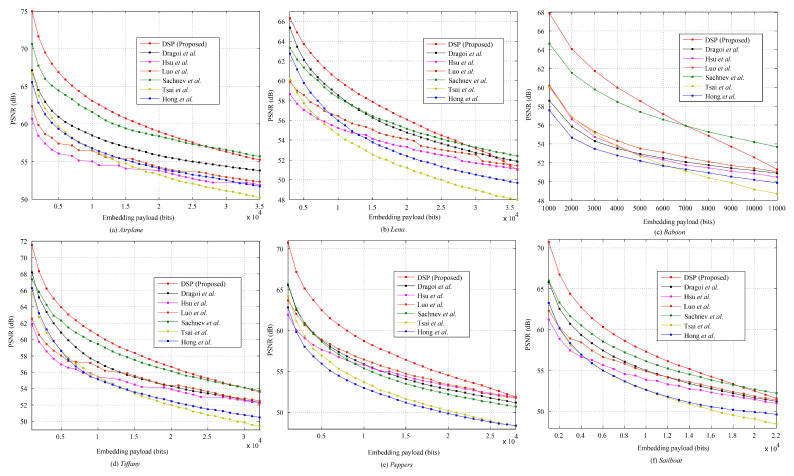
The payload-distortion performance comparison between the state-of-the-art methods of Tsai et al. [8], Sachnev et al. [10], Hong et al. [11], Luo et al. [12], Hsu et al. [23], Dragoi et al. [24], and the proposed method (DSP).

We test the above algorithm on the standard images mentioned previously. Since it is free to set *r* (please refer to Figure 5), for a payload, we generate the marked image with the highest PSNR by varying *r* from 0.01 to 0.99 with a step value of 0.01 since the data hider always has the freedom to generate a marked image with a better quality. Figure 6 shows the corresponding payload-distortion performance comparison. It is inferred that the used data hiding operation can benefit from the pixel selection and prediction procedure and, therefore, provide relatively good embedding performance. In Figure 6, with relatively low embedding rates, the proposed scheme significantly outperforms the related works. For example, for *Airplane* embedded with $0.1\times 10^{3}$ bits, the PSNR value of proposed method is 74.97 dB, which is extremely close to the theoretical uniform-embedding bound 75.33 dB (= $10\times \log_{10}\frac{255^{2}}{0.5\times 1000/512/512}$). It indicates that the proposed algorithm indeed has the ability to well-capture the smooth pixels out for data hiding. Note that, here, “*uniform-embedding*” means all PEs are shifted to carry a message that “0/1” are evenly distributed.

It can be also observed that, when the embedding payload increases, the PSNRs are likely to be relatively lower than some of the related works. For example, the proposed RDH scheme has a weaker performance for the *Baboon* image after embedded with more than $0.7\times 10^{4}$ bits. The reason is that the proposed algorithm aims to select the complex pixels for prediction and smooth pixels for data hiding, while the amount of smooth pixels within an image is actually limited due to the image content. The *Baboon* image is full of complex content so that many complex pixels are finally selected out for data hiding. However, the complex pixels are likely to be predicted with a larger PE, which, therefore, cannot keep a good payload-distortion performance. It also implies that the proposed pixel predictor should be further improved when a larger payload is required or the number of smooth pixels is limited.

**Figure 7:**
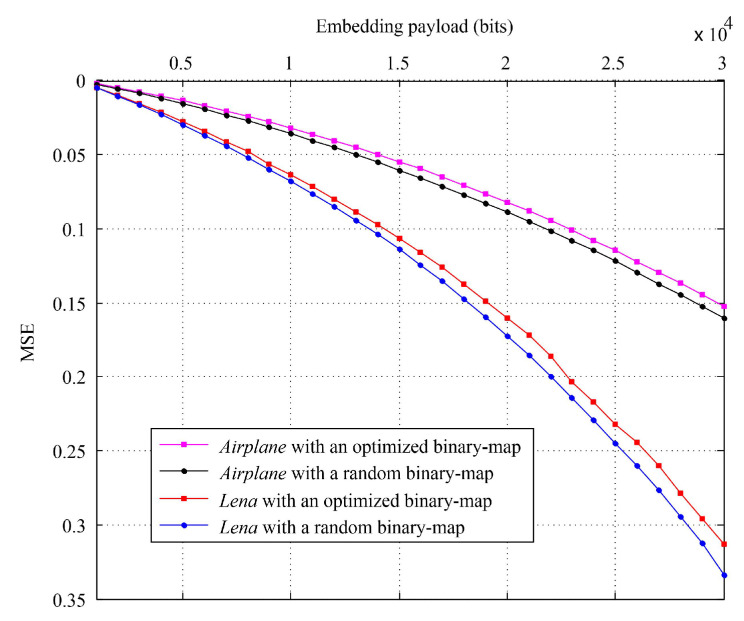
The MSE comparison between using a random binary-map (namely, using only $S_{0}$ for prediction) and using an optimized binary-map (namely, using $S_{0}\cup S_{1}$ for prediction) for the *Lena* and *Airplane* image.

In addition, to provide better performance, it is necessary to apply the optimization operation for binary-map generation (i.e., further using $S_{0}$ to generate $S_{1}$). The reason is that, for an embeddable payload, $S_{0}\cup S_{1}$ provides more original image context for pixel prediction than only $S_{0}$, which could benefit prediction accuracy. Meanwhile, the optimization operation can select regions of interest (RoI) for RDH, which can result in better payload-distortion performance. Figure 7 shows the content degradation comparison (with MSE measure) between only using $S_{0}$ and using $S_{0}\cup S_{1}$ for the *Lena* and *Airplane* image. It can be seen that an optimized binary-map would be better than a completely random binary-map in terms of payload-distortion performance.

#### 4.2.2. Evaluation on Special Images

**Figure 8:**
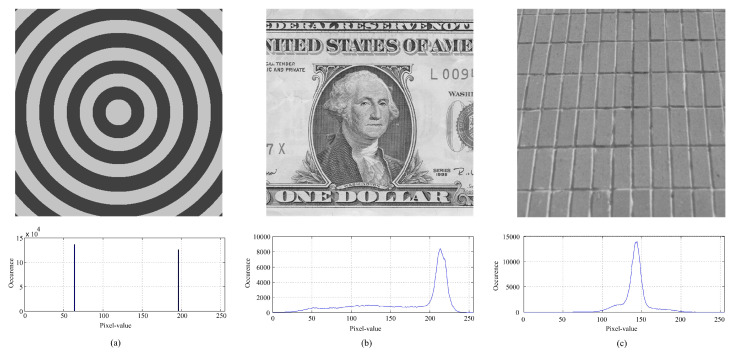
Three special test images and their histograms (all grayscaled with a size of 512 × 512): (**a**) *Circle*, (**b**) *Dollar*, (**c**) *Wall*.

**Figure 9:**
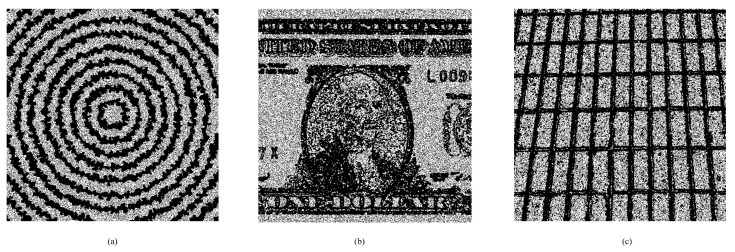
The corresponding binary-maps (r=60%) due to proposed binary-map generation procedure: (**a**) *Circle*, (**b**) *Dollar*, (**c**) *Wall*.

Unlike the traditional methods that often embed secret data according to a specified order or fully controlled by the local-complexity, the proposed DSP-based method first pre-selects relatively complex pixels out for pixel prediction and smooth pixels for data hiding. To further show its efficiency, we provide some experimental results on special images. Figure 8 shows three test images. Figure 9 shows the generated binary-maps due to the proposed binary-map generation procedure. It can be observed that the proposed method indeed can well-capture the smooth regions out for data embedding.

**Figure 10:**
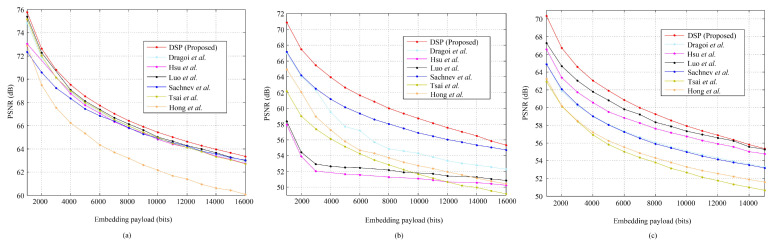
The payload-distortion performance comparison between the state-of-the-art methods of Tsai et al. [8], Sachnev et al. [10], Hong et al. [11], Luo et al. [12], Hsu et al. [23], Dragoi et al. [24], and the proposed method (DSP): (**a**) *Circle*, (**b**) *Dollar*, and (**c**) *Wall*.

**Figure 11:**
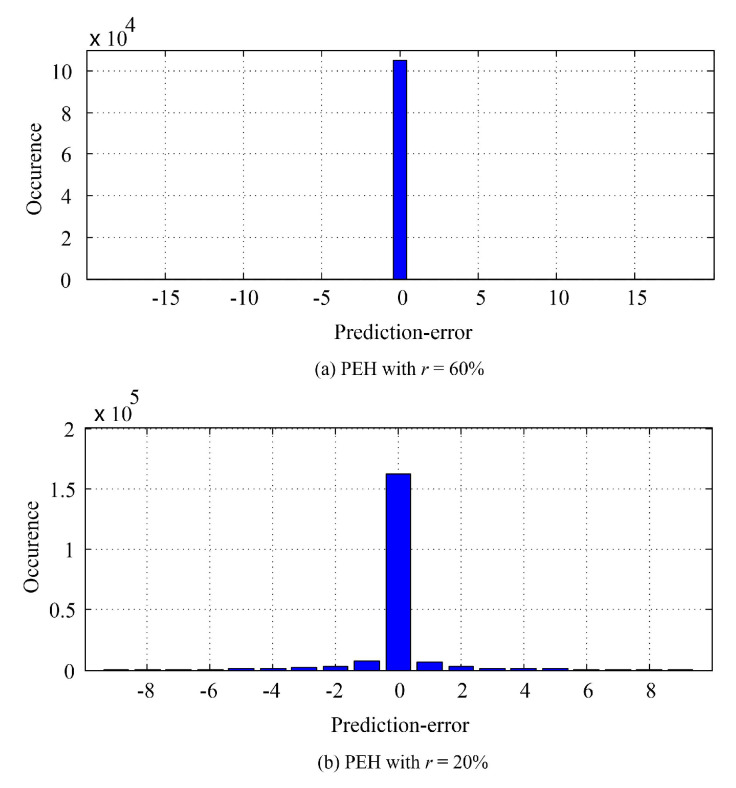
The generated PEH due to the different *r* for the *Circle* image.

We also use the special images to compare the performance between related works and the proposed method. As shown in Figure 10, the proposed method shows superior performance compared to the related works. It is worth noting that the PSNR values shown in Figure 10 of the proposed method for the *Circle* image are optimal from the viewpoint of “uniform-embedding”. For example, for a payload of $1.5\times 10^{4}$ bits, the PSNR value is 63.60 dB, while the theoretical uniform-embedding bound is $10\times \log_{10}\frac{255^{2}}{0.5\times 15000/512/512}$ = 63.57 dB. The reason is that the proposed binary-map generation procedure can select the circle-edges of the *Circle* image out such that the pixels within a connected area are all with the same value. It results in that all the prediction values are all the same as the original ones. Figure 11 shows the generated PEHs due to the different *r* for the *Circle* image. It can be observed that both the two PEHs are very sharp. Moreover, Figure 11a indicates that all the PEs of the pixels to be embedded are all with a value of zero, meaning that, for an embeddable payload, the proposed method can achieve the corresponding theoretical bound. Our experiments have shown that, even for a payload of 1.79 × 10${}^{5}$ bits, the PSNR of the proposed method for the *Circle* image is 52.41 dB, which is rather close to the theoretical bound $10\times \log_{10}\frac{255^{2}}{0.5\times 179000/512/512}$ = 52.80 dB.

## 5. Conclusions and Discussion

In this paper, we present a framework for prediction-based RDH technologies by revisiting a part of reported works. The proposed framework divides an RDH system at the data hider side into four parts so that one can design or improve an RDH system easily. We propose to use a key to generate a binary-map to improve the security, which is required in practice. Since the binary-map is always changing due to the key and the pixel prediction relies on the used binary-map, we propose to use a dynamic predictor for prediction. We also introduce a fast and efficient optimization algorithm, which can be equipped into the design of RDH or the existing works, to find the suitable HS parameters. Two novel RDH algorithms based on the proposed framework are also presented. Experimental results have shown that both the two novel RDH algorithms outperform a part of state-of-the-art works in terms of payload-distortion performance. Moreover, the proposed DSP-based algorithm can even achieve the theoretical bound of the uniform-embedding on special images. In the future, based on the proposed framework, we will focus on designing new RDH algorithms and also on improving the payload-distortion performance and the security of the existing works.

## Data Availability

Not applicable.
